# Effect of Sodium Fluoride Ingestion on Malondialdehyde Concentration and the Activity of Antioxidant Enzymes in Rat Erythrocytes

**DOI:** 10.3390/ijms11062443

**Published:** 2010-06-11

**Authors:** José A. Morales-González, José Gutiérrez-Salinas, Liliana García-Ortiz, María del Carmen Chima-Galán, Eduardo Madrigal-Santillán, Jaime Esquivel-Soto, César Esquivel-Chirino, Manuel García-Luna y González-Rubio

**Affiliations:** 1 Instituto de Ciencias de la Salud, Universidad Autónoma del Estado de Hidalgo, Ex-Hacienda de la Concepción, Tilcuautla, 42080 Pachuca de Soto, Hgo, Mexico; E-Mail: eomsmx@yahoo.com.mx (E.M.-S.); 2 Laboratorio de Bioquímica y Medicina Experimental, División de Investigación Biomédica, Centro Médico Nacional 20 de Noviembre, ISSSTE, Mexico, D.F., Mexico; E-Mail: quauhtlicutli@yahoo.com (J.G.-S.); 3 Laboratorio de Medicina Genómica, División de Medicina Genómica, Centro Médico Nacional 20 de Noviembre, ISSSTE, Mexico, D.F., Mexico; 4 Facultad de Odontologia, Universidad Nacional Autónoma de Mexico (UNAM), Mexico, D.F., Mexico; E-Mails: jaime_esquivel2003@hotmail.com (J.E.-S.); cesquivelch@gmail.com (C.E.-C.)

**Keywords:** antioxidant enzymes, erythrocyte, oxidative stress, sodium fluoride, rat

## Abstract

Fluoride intoxication has been shown to produce diverse deleterious metabolic alterations within the cell. To determine the effects of sodium fluoride (NaF) treatment on malondialdehyde (MDA) levels and on the activity of antioxidant enzymes in rat erythrocytes, Male Wistar rats were treated with 50 ppm of NaF or were untreated as controls. Erythrocytes were obtained from rats sacrificed weekly for up to eight weeks and the concentration of MDA in erythrocyte membrane was determined. In addition, the activity of the enzymes superoxide, dismutase, catalase, and glutathione peroxidase were determined. Treatment with NaF produces an increase in the concentration of malondialdehyde in the erythrocyte membrane only after the eight weeks of treatment. On the other hand, antioxidant enzyme activity was observed to increase after the fourth week of NaF treatment. In conclusion, intake of NaF produces alterations in the erythrocyte of the male rat, which indicates induction of oxidative stress.

## 1. Introduction

Fluoride is a very abundant chemical element on the surface of the earth and is recognized as an element that may produce chronic alterations in humans when they are exposed to it. Fluorosis (intoxication by fluoride) is caused by acute or chronic intake of fluorides and is clinically characterized in humans by means of alterations in dental cavities, as well as by changes in the musculo-skeletal and nervous systems [1–3].

*In vivo* and *in vitro* studies have recognized that intoxication with fluoride may produce diverse metabolic alterations within the cell, such as inhibition of glycolysis, alteration in membrane receptors, changes in total energetic balance, DNA rupture, and induction of apoptosis [4–7]. Because study models of fluoride exert deleterious effects on the organism, intoxication models have been developed with sodium fluoride (NaF), which is administered to experimental animals by several means, doses and time actions. Among NaF intoxication models, those utilizing the compound by adding it to drinking water of experimental animals are prominent [4–8]. These models present several methodological advantages, among which are noteworthy includes: easy application of the toxic substance (in drinking water) and care of required doses, as well as the ease of control and monitoring of the treated animals.

Our work group has reported that ingestion during four weeks of 50 ppm of NaF included in drinking water for rats produces oxidative stress in the oral mucosa, characterized by an increase in the levels of malondialdehyde (a marker of lipoperoxidation), as well as alterations in the enzymes superoxide dismutase and catalase [8].

Oxidative stress is developed when there is an increase in the production of free-radical derivatives from oxygen or when antioxidant systems are unable to contain the former [8–12].

On the other hand, the erythrocyte is one of the main cells used as an oxidative stress marker in living animals, including humans, because their cell membranes, as well as their antioxidant enzymes, are sensitive to the presence of free radicals in general, as well as in local levels [11,13,14]. Furthermore, access by venipuncture to this type of cell entails a methodological advantage over obtaining samples with very little damage to tissues.

Because it has been demonstrated that the ingestion of NaF added to drinking water induces oxidative stress in the oral mucosa of the rat, the objective of the present work was to determine the effect exerted by the ingestion of this toxin on the concentration of malondialdehyde and on the activity of antioxidant enzymes, superoxide dismutase, catalase, and glutathione peroxidase, in erythrocytes of the rat as an example of the general oxidative stress induced by this toxin.

## 2. Materials and Methods

### 2.1. Chemicals and Animals

Male Wistar rats (total, 50 animals) with an average body weight of 240 ± 7 g were obtained from Harlam Laboratories (Harlam de México, S.A. de C.V.) and placed, for their acclimatization, in individual containers with 12 h light-dark cycles and with free access to food and water for 1 week. The fluoride content in the food (Purina Pellet Chow; Purina, St. Louis, MO, USA) and the drinking water do not interfere with the treatment applied because the average amount of F in each case is <0.3 ppm [1,6,8,15]. All procedures were carried out according to the guidelines contained in the Regulation on Treatment of Animals for Surgery and Research of our institution, which is in agreement with the Federal Regulation Law for Research and Experimentation Animals (SAGAR, Mexico, 1999).

Chemical reagents used were of analytical grade and of the best possible quality. They were obtained from Sigma Chemical Co. (St. Louis, MO, USA), Merck de México S.A., or from Mallinckrot de México, S.A.).

### 2.2. The Treatment Protocol

After the acclimatization week, the animals were weighed and randomly divided, according to the protocols already established [1,6,8,15,16], into two study groups (25 animals per group). The two study groups were:

- Control: Rats that ingested food and water in a free manner with no type of treatment;
- NaF (Experimental): Rats with free access to food and whose drinking water contained NaF.

Once the treatment began, food and water were provided daily. The daily consumption of both groups were registered and then summarized weekly.

The animals were observed during the entire treatment time to detect signs of fluorosis (e.g., piloerection, alopecic zones, decrease in motor activity, and speckled yellow teeth) according to already established criteria [1,6,15–17].

Rats of both groups were sacrificed by decapitation every 2 weeks (five animals per group) for up to 8 weeks. These were examined carefully for signs of fluorosis.

Blood was collected in tubes with anticoagulant (EDTA) and centrifuged at 2,000 rpm for 15 min in a clinical centrifuge. Plasma and leukocytes were discarded, and three volumes of cold, sterile phosphate-buffer saline (PBS: 0.9% NaCl in 0.01 M phosphate buffer pH 7.0) were added; this was centrifuged at 2,000 rpm for 15 min. In accordance with previously described techniques, the package of erythrocytes was washed twice more and resuspended in PBS to obtain a hematocrit of 50% [13,14,18].

### 2.3. Obtention of Cytosolic Fraction and Cell Membranes

The obtained, erythrocytes were lysed, employing a hypotonic phosphate buffer (5P8; phosphate buffer 5 mM, pH 8), according to previously reported techniques [13,14,18]. The procedure is as follows: a volume of erythrocyte fraction was washed with 5P8 for 15 min at 4 °C. The result was centrifuged at 10,000 rpm for 15 min. The recovered supernatant was considered the cytosolic fraction, which was used to determine antioxidant enzyme activity.

On the other hand, the precipitate was considered the cell membrane fraction and was washed twice more with 5P8 and suspended in isotonic saline solution (NaCl 0.9%) until its later use.

### 2.4. Malondialdehyde Determination

The malondialdehyde concentration (MDA), as the index of lipoperoxidation, was determined in the membranes of the erythrocytes according to the method described by Gutiérrez *et al*. [8,13] that is based on that of Ohkawa *et al*. [19]. A sample of erythrocyte membranes (300 mg of total protein) was incubated with 1 mL of Tris-Buffer (Tris-HCI, 0.15 M, pH 7.0) at 37 °C for 30 min. At the end of the incubation, 1.5 mL of acetic acid (5% v/v) and 1.5 mL of thiobarbituric acid (0.8% p/v) were added and this mixture was incubated for 60 min at 90 °C. At the end of incubation, we added 1 mL of KCL (2% p/v), 3 mL of a mixture of butanol/pyridine was recovered, and protein determination was performed using a spectrophotometer (Jenway 6300, Cielo Vista, CA, USA); the absorbance λ was set at 532 nm. MDA concentration was calculated with extinction coefficient (1.54 × 10^5^ M^−1^ cm^−1^) and expressed as nmol/(L*mg) of total protein.

### 2.5. Determination of Antioxidant Enzyme Activity

In the cytosolic fraction of the erythrocytes, we determined the specific activity of the antioxidant enzymes superoxide dismutase, catalase, and glutathione peroxidase, according to the following described procedures. Prior to determination of the activity of the antioxidant enzymes, we eliminated excess hemoglobin according to the method of Bannister and Bannister [20]. The following procedure was utilized: one volume of cytosolic fraction was mixed with one volume of ethanol/chloroform (5/3, v/v) with continuous agitation for 10 min, and then 1/5 volume of isotonic NaCl was added before. The result was centrifuged at 3,000 rpm for 60 min and the precipitate discarded. The supernatant was considered the hemoglobin-free cytosolic fraction (FCsHb), which was used for determination of enzyme activity. The activity of the Superoxide dismutase enzyme (SOD) was determined according to the technique reported by McCord and Fridovich [21], by means of the formation of formazan from the chloride compound of 2-(4-Iodophenyl)-3-(4-nitrophenyl)-5-phenyltetrazolium chloride (INT).

The procedure was as follows. A sample of FCsHb was diluted 10 times with phosphate-buffer (KH_2_P0_4_, 10 mM, pH 7.0), and then 170 μL of reaction mixture (Xantine, 0.05 mM; INT, 0.025 mM) were added. The reaction was started by adding 25 μL of Xantine oxidase (80 U/L) and 10 μL of H_2_0_2_ (3 %/water). The absorbance was monitored at 500 nm for 210 s. The specific activity was reported in μmol/(min*mg of protein) [22].

Catalase activity was determined according to the procedure reported by Aebi [22]. A sample of FCsHb was incubated for 5 min with one volume of PBS at 37 °C. The reaction was started by adding 10 mM of H_2_O_2_ and followed for 45 s at 240 nm. The resulting activity was expressed in μmol /(min*mg of protein).

The specific activity of the enzyme glutathione peroxidase (Glpx) was determined using the Glutathione peroxidase cellular activity assay kit from Sigma Aldrich, St. Louis, MO, USA, following the manufacturer’s instructions and based on the method of Paglia and Valentine [23]. Enzyme activity was reported in μmoles/(min*mg of protein). The amount of total protein in a membrane fraction, as well as in the FCsHb, was determined according to the standard procedure of Lowry [24], utilizing bovine serum albumin as the standard.

### 2.6. Statistical Analysis

Results are expressed as average values ± Standard error (SE) and were analyzed employing the GraphPad Prism V-4.00 statistical program (San Diego, CA, USA). The mean difference between groups was made by the Student *t* test. A probability level of <0.05 was taken as significant *(p* < 0.05).

## 3. Results

Table 1 summarizes the weight gain data for the control rats and NaF-treated rats, along with their weekly ingestion of water and food. As can be observed, there was no significant difference between the weight gain between the two groups (156.9 ± 6.2 *vs.* 157.9 ± 5.4; control *vs.* NaF, respectively; p > 0.5). On the other hand, there were also no statistically significant differences between the amount of water or food that the rat consumed weekly.

After calculating the amount of water consumed by the rats, the amount of NaF ingested monthly by the treatment group was calculated (171.44 mg of NaF/kg of body weight).

Some signs of fluorosis were perceived in the data that were in accordance with the clinical parameters that were employed and previously reported to define intoxication by fluoride in a rat [1,6,8,15–17] and with the calculated doses that the animals received during eight weeks.

Table 2 depicts MDA concentration in the erythrocyte membrane throughout the treatment of in both groups. As can be noted, the MDA concentration presents a significant statistical increase (p < 0.5) in rats treated with NaF in comparison with the control group, only in the eighth week of treatment. Because MDA is a product of lipoperoxidation of the fatty acid of the cellular membrane, and because this phenomenon presents itself when there is an excess of free radicals, we are able to suppose that the erythrocyte membrane has been damaged by these reactive species. These species were induced by intoxication by means of NaF.

The specific activity of the main antioxidant enzymes of the erythrocyte throughout the eight weeks of treatment with NaF is observed in Table 3. This shows the activity of the SOD enzyme deriving from the cytosol of the erythrocytes of NaF-treated rats, as well as their respective control group. As with MDA, SOD activity presents a statistically significant increase in comparison with the control group, but this time, after only the fourth week of treatment. This increase becomes ever more pronounced, rising up to 66% (with respect to the control group) after eight weeks of NaF treatment.

Catalase and Glpx derived from the erythrocytes of NaF-treated rats also showed a statistically significant increase in their specific activity after four weeks of NaF treatment, with, an increase equivalent to 135% of the specific activity of catalase produced, with respect to the control group. The increase in the activity of this enzyme rose to 218 and 298% at six and eight weeks, respectively (Table 3). On the other hand, the specific activity of Glpx shows an increase equivalent to 33, 41, and 53% with respect to the control group at four, six, and eight weeks of treatment with NaF (Table 3).

## 4. Discussion

As has been previously reported by our work group, intoxication with doses of 50 ppm of NaF applied in drinking water to rats for at least a period of four weeks does not produce the appearance of clinical data of fluorosis [8]. However, rats treated for up to eight weeks with this amount of the toxin, according to the results presented in this work, do exhibit some intoxication data due to fluorides.

It is characteristic of rats that are induced with fluorosis, by means of continuous ingestion of NaF in their drinking water, to present signs such as lack of gain of body weight (due in part to a decrease in food intake), together with the presence of piloerection, alopecic zones, decrease in general motor activity, and yellow staining of the teeth. These signs of fluorosis are generally present in animals that have been placed under ingestion of NaF for >12 weeks in doses ranging from 100–800 ppm [1,6,15–17].

The data shown in Table 1 indicates that animals treated with 50 ppm of NaF for up to eight weeks do not present changes in relation with the control group, in terms of gain of total body, as well as in weekly consumption of food and water. On the other hand, the meticulous observation of the animals to detect piloerection, alopecic zones, yellow stains in teeth, and decrease in general motor activity, indicates that some of these signs of fluorosis were found. Thus, we are able to ensure that our treatment is capable of inducing “clinical fluorosis”, as has been described for other experimental models [1,6,15–17]. As we cited in a previous article [8], it is probable that the time of treatment that we have used (8 weeks) is not sufficient to produce a general level of visible alterations from the fluorosis; nevertheless, the changes found in the concentration of MDA, as well as in the antioxidant enzymes, show us that NaF is able to induce metabolic changes in the erythrocyte produced by our treatments. Although this does not translate into “clinically visible” alterations, it denotes an effect, produced by this toxin, on the antioxidant systems of the erythrocyte.

It has been indicated that alteration in the specific activity of antioxidant enzymes in the cell or tissues, in addition to an increase in lipoperoxidation (determined by an increase in MDA) of the fatty acid of cellular membranes, are indicative of oxidative stress, which is mainly characterized by an excessive production of free radicals derived from oxygen or a decrease in antioxidant mechanisms [8–10,25].

It has been reported that acute or chronic intoxication with NaF in experimental animals, as well as in humans who live in zones of fluorosis, produces oxidative stress in several organs, such as the kidney, brain, liver, and gonads. In such subjects, the clinical signs of fluorosis appear accompanied by alterations in the enzyme activity of SOD, catalase, and Glpx, in addition to an increase in MDA levels in the serum or fatty acids of the tissues [26–28]. The mechanism by which NaF may induce oxidative stress in these organs is not known with certainty.

Nevertheless, studies conducted *in vitro* show that NaF may interact with enzymes that contain a transition metal as part of their co-factors or in their active site. Fluoride, by virtue of its chemical nature, is capable of interrelating with metal and thus can exert activating or inhibitory influences upon enzyme activity [26–28]. The SOD, catalase, and Glpx enzymes, which are the main antioxidant enzymes of the erythrocyte, present a transition metal as a co-factor; thus, the interaction study of this toxin with these enzymes may explain the behavior that the enzymes exhibit *in vivo*. On the other hand, our results show that the erythrocyte membrane presents an increase of MDA only after eight weeks of treatment with NaF, which contrasts with studies reporting an increase of these metabolites after two weeks of treatment with NaF [1–3,7,26–29]. However, the amount of NaF employed (250 to 1,000 ppm) may be a factor for obtaining a diversity of results. Additonally, the application pathway (intramuscular or intraperitoneal), can also affect the results; the same amount of NaF employed can have different effects in accordance with the pathway used.

As has been shown, an increase in MDA values are indicative of oxidative stress induced by free radicals derived from oxygen; nevertheless, it has been demonstrated that the increase in MDA levels are possible only when the antioxidant defenses provided by the enzymes have been overcome [8–11]. In this fashion, the damage caused by free radicals in the cell membrane with the consequent production of MDA is possible when the main antioxidant enzymes that the cell possesses, mainly in? the erythrocyte, cannot “contain” these antioxidants and they “escape” to damage the fatty acids, giving rise to an increase in MDA levels.

The results obtained here allow us to suppose that it is not until eight weeks of treatment with NaF that this toxicant exercises its harmful action on the cellular membrane, because from four weeks of exposure to the toxicant, the activity of the SOD, catalase, and Glpx antioxidant enzymes presents changes in their specific activity, as if there were a “preamble,” in which oxygen-derived free radicals, by intoxication with NaF, are inside the antioxidant enzymatic systems possessed by the erythrocyte. At eight weeks of treatment with the toxin, the antioxidant enzyme activity of the erythrocyte is at the maximum and probably does not counterbalance the production of free radicals; thus, damage to the cell membrane is presented with the subsequent increase in MDA concentration.

With all that has been presented previously herein, we are able point out that the erythrocyte of the rat is metabolically sensitive to the presence of NaF, and that this sensitivity is clear, due to the changes that are observed in the activity of the antioxidant enzymes SOD, catalase, and Glpx. The previous data, along with the increase in MDA after eight weeks of treatment, demonstrate to us the presence of NaF-induced oxidative stress.

## 5. Conclusion

The effect of NaF on the erythrocytes that we have observed in this study permits us to suppose that one of the psychopathological mechanisms of damage caused by this toxin is probably through the alteration of antioxidant enzymes that are no longer able to contain damage to the cell membrane, originating as an increase in the levels of MDA, which altogether can be interpreted as induction to oxidative stress.

It is clear that additional research is required to evaluate more precisely the effects, mainly in humans, that NaF may have on the antioxidant systems of the erythrocyte. Thus, future studies directed toward solving this type of questioning are very important and should be developed.

## Figures and Tables

**Table 1.** General characteristics of rats that received 50 ppm of NaF supplemented in their drinking water for eight weeks and untreated control animals.

**Group**	***N***	**CSAI (g)**	**CSAg (mL)**	**PI (g)**	**PF (g)**	**GPF (g)**
**Control**	25	145.3 ± 8.1	208.3 ± 3.5	253.5 ± 4.2	410.4 ± 3.2	156.9 ± 6.2
**NaF**	25	153.2 ± 7.2	219.5 ± 5.4	250.3 ± 4.4	408.2 ± 4.5	157.9 ± 5.4

Results are expressed as average ± Standard error of mean (SEM). Study groups correspond to eight weeks of treatment. CSAI: weekly food consumption; CSAg: weekly water consumption; PI: beginning body weight; PF: final body weight; GPF: Gain of body weight.

**Table 2.** MDA concentration (nmoles/mg total protein) in membranes of erythrocytes from rats treated with NaF for up to 8 weeks and control rats.

	**MDA (nmoles/mg protein)**	
Time (weeks)	Control	NaF
0	289.35 ± 25.3	294.54 ± 32.11
2	294.54 ± 22.01	311.87 ± 22.31
4	275.54 ± 24.02	266.61 ± 24.31
6	280.30 ± 24.21	309.26 ± 23.02
8	265.40 ± 23.02	375.36 ± 25.31*

Results are the average ± Standard error of mean (SEM) of five rats per week.

* < 0.05 *vs.* control.

**Table 3.** Activity of erythrocyte antioxidant enzymes from rats treated with NaF for up to 8 weeks and control rats.

	**SOD (μmol/min/mg protein)**		**Catalase (μmol/min/mg protein)**		**Glutathione peroxidase (μmol/min/mg protein)**	
Time (weeks)	Control	NaF	Control	NaF	Control	NaF
0	217.23 ± 10.23	215.06 ± 11.02	15.86 ± 3.50	15.92 ± 3.02	56.41 ± 4.02	57.31 ± 4.23
2	184.01 ± 12.61	223.66 ± 12.51	15.79 ± 4.01	26.42 ± 3.22	63.44 ± 3.24	63.34 ± 4.36
4	209.25 ± 16.81	256.61 ± 16.25*	15.54 ± 3.22	47.52 ± 5.23*	55.31 ± 3.74	78.34 ± 3.75*
6	217.23 ± 17.32	301.41 ± 20.32*	15.81 ± 3.56	60.72 ± 5.42*	59.34 ± 4.23	83.34 ± 3.84*
8	207.23 ± 17.23	357.32 ± 17.23*	15.72 ± 4.21	83.32 ± 6.95*	61.42 ± 3.74	89.25 ± 4.54*

Results are the average ± Standard error of mean (SEM) of five rats per week.

* <0.05 *vs.* control.
